# Establishment and characterization of a new mantle cell lymphoma cell line with a NOTCH2 mutation, Arbo

**DOI:** 10.1002/jha2.580

**Published:** 2022-09-20

**Authors:** Firas Safa, Terri Rasmussen, Patricia Lobelle‐Rich, Stephanie Collier, Nicholas Milligan, John Schmeig, Janet Schmid, Carol Wiewiorowski, Denise Totaro, Theresa C. Brown, Ishwarya Satyavarapu, Melody Badoo, Nathan Ungerleider, Erik K. Flemington, Hana Safah, Nakhle S. Saba

**Affiliations:** ^1^ Section of Hematology and Medical Oncology, Deming Department of Medicine Tulane University New Orleans Louisiana USA; ^2^ Department of Pathology Children's Hospital New Orleans Louisiana USA; ^3^ Department of Pathology Tulane University New Orleans Louisiana USA; ^4^ Hayward Genetics Center Tulane University New Orleans Louisiana USA; ^5^ Tulane Cancer Center Tulane University New Orleans Louisiana USA

**Keywords:** CD23, cell line, mantle cell lymphoma, NOCTH2

## Abstract

Cell lines represent an essential tool used in preclinical research. Most hematologic malignancies have a wide array of cell lines representing their respective molecular and pathologic spectra. In mantle cell lymphoma (MCL), cell lines become specifically valuable in view of the heterogeneity of this disease. Unfortunately, the number of MCL cell lines that are available for the research community remains small, with only nine cell lines available for purchase through the American Type Culture Collection (ATCC). We have established a novel blastoid MCL cell line, isolated from the malignant pleural effusion of a 69‐year‐old male with refractory MCL. Arbo was fully characterized with cytogenetics, immunophenotyping, whole exome sequencing and drug sensitivity assays. One of the most notable mutations identified in Arbo (but not in normal tissue) was the missense mutation NOTCH2 R2400*, which has been proposed as a clinically significant mutation in MCL seen in 5% of cases. NOTCH2 R2400* results in a truncated Notch2 protein, leading to a more stable and active protein. Using pharmacologic inhibition of Notch2, we showed a dependence of Arbo on NOTCH2 signaling, as well as a link between CD23 expression on Arbo and NOTCH2 activity. Arbo represents a NOTCH2 mutated model that is useful in MCL as well as other lymphomas with such mutation. We plan to deposit Arbo at the ATCC to be available for the research community.

1

Mantle cell lymphoma (MCL) is an aggressive, largely incurable, and subtype of non‐Hodgkin's lymphoma (NHL) that commonly involves the peripheral blood, bone marrow, and lymphatic system [1]. The genetic hallmark of MCL is the chromosomal translocation t(11;14)(q13;q32), resulting in cyclin‐D1 overexpression. MCL is a rare disease, which accounts for ∼6% of NHL but disproportionately contributes to NHL‐related mortality. Preclinical research studies, classically based on patient‐derived samples, face significant challenges due to limited availability of such samples. Additionally, although cell lines represent a very useful tool for cancer research, there are only a few that have been fully characterized and are currently used in MCL research. A search for MCL cell lines in the Cellosaurus database [2] revealed only 10 cell lines available for purchase through the American Type Culture Collection (JMP‐1, JeKo‐1, REC‐1, JVM‐2, JVM‐13, Mino, PF‐1, MAVER‐1, Z‐138, and NCEB‐1). Unfortunately, this cell line menu is relatively old and does not cover the wide spectrum of MCL molecular and biologic heterogeneity. Recent advances in MCL unveiled roles for novel genetic alterations with potential therapeutic applications. In a recent study, Bea et al. used next generation sequencing on MCL primary samples as well as six MCL cell lines to investigate for recurrent genetic alterations. Most encountered mutations involved ATM, CCND1, and TP53 that have been previously described, as well as mutations in WHSC1, MLL2, BIRC3, MEF2B, TLR2, and NOTCH2. Some of these mutations such as MLL2 and MEF2B are represented in MCL cell lines (MLL2 in JEKO and JVM2, and MEF2B in REC). However, NOTCH2‐activating mutations, namely R2400*, observed in 5.2% of patients and negatively impacting their prognosis, remain without a representative cell line [3].

Notch2 is a transmembrane protein that plays a critical role in B‐cell maturation throughout most of its lifespan [4]. Notch2 is activated following cleavage and release of its intracellular domain in the cytoplasm followed by its translocation into the nucleus where it induces target genes transcription. At the end of its life cycle, Notch2 is inactivated by phosphorylation and subsequent ubiquitination leading to degradation by the proteasome. The C‐terminal PEST domain is essential for the deactivation process; mutations in this domain, such as R2400*, interfere with this process, thus resulting in enhanced stability (and subsequently activity) of Notch2 [3, 4].

Here we present Arbo, a recently established *NOTCH2* R2400*‐mutated blastoid MCL cell line, isolated from a malignant pleural effusion of a patient with MCL.

Arbo was established from a 69‐year‐old male initially hospitalized for fatigue and weight loss. His physical exam was significant for massive hepatosplenomegaly along with cervical, abdominal, and pelvic lymphadenopathy. Blood counts revealed normocytic anemia with a hemoglobin of 9 g/dl and a significant lymphocytosis at 19,700 × 10^6^/ml. A bone marrow biopsy and aspirate showed a prominent population of kappa‐restricted B cells with immunohistochemical findings consistent with a diagnosis of MCL. Tumor cells were positive for CD5, CD20, CD79a, FMC7, CD22, and cyclin‐D1, and negative for CD3, CD23, CD200, and CD11c. Cells expressing the proliferation marker Ki‐67 represented 90% of total. Fluorescence in situ hybridization (FISH) confirmed the presence of translocation t(11;14)(q13;q32) associated with MCL. FISH also detected the deletion of one copy of 13q14 in 69% of cells, and of both 13q14 in 13% of cells.

The patient's *Mantle Cell Lymphoma International Prognostic Index* (MIPI) score was 10.1, indicating a high‐risk disease. He initially received one cycle of rituximab, cyclophosphamide, vincristine, doxorubicin, and dexamethasone alternating with rituximab, high‐dose methotrexate, and cytarabine (R‐hyperCVAD/R‐MTX/Ara‐C), resulting in a rapid clinical response. The treatment was switched to bendamustine plus rituximab due to intolerance, and the patient subsequently completed six cycles resulting in a complete remission. Shortly after the initiation of maintenance therapy with rituximab, the patient suffered a relapse with a malignant left‐sided pleural effusion, splenomegaly, and pancytopenia. Interestingly, immunophenotyping of the pleural fluid performed at the time of relapse showed a heterogeneous expression of CD23. He was treated with R‐CHOP (rituximab, cyclophosphamide, doxorubicin, vincristine, and prednisone) but unfortunately progressed and succumbed to his disease.

At relapse, with informed consent, a sample of the malignant pleural effusion was obtained, and mononuclear cells were isolated by Ficoll density gradient centrifugation. Following a few days of in vitro culture in RPMI 1640 supplemented with 10% fetal bovine serum and antibiotics (penicillin and streptomycin at 5%) at 37°C with 5% CO_2_, cells started to grow rapidly requiring culture medium support without the need for cytokines, resulting in an estimated doubling time of 48 h. The cells were able to survive multiple freeze/thaw cycles and were maintained in continuous culture for more than 12 months.

To determine whether Epstein Barr Virus (EBV) played a role in Arbo's immortalization, qPCR was performed using EBV‐specific primers on DNA extracted from Arbo and primary MCL cells that had been purified from the malignant pleural effusion and selected by CD19+ on‐column selection using magnetic beads. Both Arbo and the primary MCL cells were negative for EBV DNA, using appropriate controls.

To evaluate for possible changes associated with the immortalization process, we compared Arbo to the original tumor using flow cytometry and cytogenetic testing. Both samples showed kappa‐restricted cells positive for CD5, CD19, CD20, CD22, and CD23, and negative for CD2, CD3, CD7, CD10, and CD71. Two differences were detected: FMC7 that was positive in Arbo (100%) and negative in primary cells (6%), whereas CD38 was negative in Arbo (0%) and positive in the primary cells from the pleural effusion (97%) (Figure S1). This potentially indicates antigenic modulation involving CD38 and FMC7, during the adaptation of Arbo to the cell culture microenvironment [5]. With regard to cytogenetic testing, Arbo was found to have a complex karyotype (51, XY, +1, add(2)(p11.2), +7, add(8)(p11.2), −10, add(11)(p11.2), t(11;14)(q13;q32),+add(13)(q34), del(13)(q14q14)x1‐2, add(18)(p11.2),−22,+r, +1, −3mar[cp22]) (Figure 1A). The presence of the translocation t(11;14)(q13;q32) was confirmed by FISH in both Arbo and the primary tumor (Figure 1B). Additionally, the overexpression of cyclin‐D1 by Arbo was confirmed by western blot using appropriate positive (Jeko and UPN1) and negative (RCH‐ACV) controls (Figure 1C).

**FIGURE 1:**
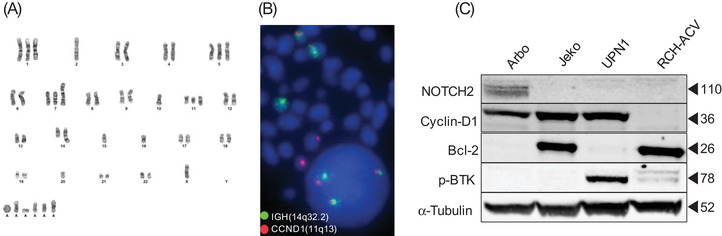
Arbo originated from a subset of the original mantle cell lymphoma (MCL) tumor cells. (A) Arbo's complex karyotype: (51, XY, +1, add(2)(p11.2), +7, add(8)(p11.2), −10, add(11)(p11.2), t(11;14)(q13;q32), +add(13)(q34), del(13)(q14q14)x1‐2, add(18)(p11.2), −22, +r, +1, −3mar[cp22]). (B) Fluorescence in situ hybridization (FISH) detecting the t(11;14)(q13;q32). The probe for the *IGH* gene (14q32.2), illustrated by the green dot, and the probe for the *CCDN1* gene (11q13), illustrated by pink. (C) Immunoblot for Notch2, cyclin D1, Bcl‐2, and pBTK in Arbo, Jeko, UPN‐1, and RCH‐ACV

To further investigate Arbo's complex karyotype and characterize its genetic makeup, we performed whole exome sequencing (WES) on DNA collected from Arbo and the primary tumor cells. WES revealed a total of 143,761 single nucleotide polymorphisms in Arbo, which included 10,286 missense mutations, 104 stop‐gain, 33 stop‐loss, 18 start‐loss, and 84 splicing mutations (Figure S2). Key mutations include *ATM* 5557G > A (p.D1853N) and 5948A > G (p.N1983S), *TP53* 215C > G (p.P72R) and 617T > A (p.L206*), and *NOTCH2* 7198C > T (p.R2400*) (a complete list of the mutations found on WES with their corresponding variant allele frequency is provided in the Supporting Information section).

To investigate whether NOTCH2 R2400* results in increased protein stability, we compared Notch2 expression in Arbo to other cell lines. Indeed, our western blot confirms the overexpression of Notch2 in Arbo (Figure 1C). Furthermore, NOTCH2 R2400* was confirmed to be somatic as it was detected in DNA extracted from Arbo but not in normal tissues (uninvolved kidney sample) using Sanger sequencing (Figure 2A).

**FIGURE 2:**
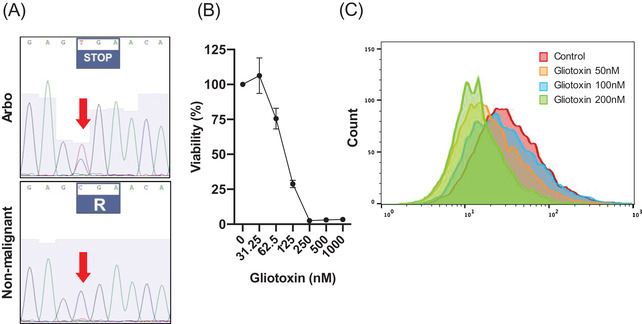
Detecting and evaluating the impact of the NOTCH2 R2400* mutation in Arbo. (A) Sanger sequencing performed to show presence of a STOP codon in Arbo cells, which was not present in kidney cells of our patient. (B) Dose‐dependent decrease in Arbo's viability following a 48‐h exposure to gliotoxin. (C) Changes in CD23 expression in Arbo's cells, measured by flow cytometry, following exposure to increasing concentrations of gliotoxin

To determine Arbo's dependence on Notch2 for survival, we cultured Arbo with increasing concentrations of gliotoxin, a Notch2 transactivation inhibitor [6]. Following 48‐h incubation with gliotoxin, we detected a dose‐dependent decrease in cell viability using a colorimetric assay, resulting in an IC_50_ of ∼100 nM.

Classically, MCL cells lack expression of the B‐cell differentiation/activation marker CD23, a pattern used to differentiate MCL from chronic lymphocytic leukaemia (CLL). However, a subset of MCL expresses CD23 with ill‐defined causes and consequences. Additionally, the expression of CD23 has been highly associated with Notch2 activity [7]. To determine whether CD23 expression on Arbo is controlled by Notch2 signaling, we quantified CD23 expression by flow cytometry following Notch2 inhibition by serial concentrations of gliotoxin. As expected, we observed a dose‐dependent downregulation of CD23 expression following Notch2 inhibition confirming the relationship between the two.

While in CLL, Notch2 overexpression is linked to in vitro resistance to the Bcl‐2 inhibitor venetoclax [8], the association of which in MCL remains unknown. To determine Arbo's sensitivity to Bcl‐2 inhibition, we treated Arbo cells with increasing concentrations of venetoclax for 72 h and found that the cells are highly resistant and can withhold concentrations up to 4 µM without significant cell death (Figure S3).

In contrast, Arbo seems to depend on B‐cell receptor (BCR) signaling for survival, as they were sensitive to BTK inhibition with ibrutinib, with a 48‐h IC_50_ of ∼2 µM (Figure S3). Dependence on BCR signaling was also confirmed by the detection of pBTK by immunoblot in unstimulated cells (Figure 1C). In conclusion, Arbo is a new blastoid NOTCH2 mutated MCL cell line that is well characterized, and that can serve as a model for Notch2 signaling in MCL. Arbo is planned to be deposited in ATCC to be available to the research community.

## AUTHOR CONTRIBUTIONS

Firas Safa and Terri Rasmussen acquired data, interpreted the results, and drafted and revised the manuscript. Patricia Lobelle‐Rich, Stephanie Collier, Nicholas Milligan, Theresa C. Brown, Ishwarya Satyavarapu, Melody Badoo, and Nathan Ungerleider acquired data, reviewed, and approved the final version of the manuscript. Denise Totaro and Carol Wiewiorowski acquired data. Janet Schmid, John Schmeig, Erik K Flemington, and Hana Safah reviewed and approved the final version of the manuscript. Nakhle S. Saba conceived and designed the work, acquired data, interpreted the results, and drafted and revised the manuscript.

## CONFLICTS OF INTEREST

The authors declare they have no conflicts of interest.

## ETHICS STATEMENT

The study received the IRB approval and appropriate guidelines were followed.

## PATIENT CONSENT STATEMENT

Samples were collected from the patient following written consent.

## Supporting information

Supplement Material

Supplement Material

## Data Availability

The data that support the findings of this study are available in the Supporting Information of this article.

## References

[1] Saba N , Wiestner A . Do mantle cell lymphomas have an “Achilles heel”? Curr Opin Hematol. 2014;21(4):350–7. 10.1097/MOH.0000000000000057 24857884 PMC4144918

[2] Bairoch A . The Cellosaurus, a cell‐line knowledge resource. J Biomol Tech: JBT. 2018;29(2):25–38. 10.7171/jbt.18-2902-002 29805321 PMC5945021

[3] Bea S , Valdes‐Mas R , Navarro A , Salaverria I , Martín‐Garcia D , Jares P , et al. Landscape of somatic mutations and clonal evolution in mantle cell lymphoma. Proc Natl Acad Sci USA. 2013;110(45):18250–5. 10.1073/pnas.1314608110 24145436 PMC3831489

[4] Amsen D , Deaglio S , Arruga F , Vaisitti T . The NOTCH pathway and its mutations in mature B cell malignancies. Front Oncol. 2018;8:550. 10.3389/fonc.2018.00550. www.frontiersin.org 30534535 PMC6275466

[5] Onciu M , Berrak SG , Medeiros LJ , Katz RL , Huh YO . Discrepancies in the immunophenotype of lymphoma cells in samples obtained simultaneously from different anatomic sites. Am J Clin Pathol. 2002;117(4):644–50. 10.1309/URTD-7MD9-U8N1-C60Q 11939741

[6] Hubmann R , Hilgarth M , Schnabl S , Ponath E , Reiter M , Demirtas D , et al. Gliotoxin is a potent NOTCH2 transactivation inhibitor and efficiently induces apoptosis in chronic lymphocytic leukaemia (CLL) cells. Br J Haematol. 2013;160(5):618–29. 10.1111/bjh.12183 23278106

[7] Hubmann R , Düchler M , Schnabl S , Hilgarth M , Demirtas D , Mitteregger D , et al. NOTCH2 links protein kinase C delta to the expression of CD23 in chronic lymphocytic leukaemia (CLL) cells: Research paper. Br J Haematol. 2010;148(6):868–78. 10.1111/J.1365-2141.2009.08024.X 19995395

[8] Fiorcari S , Maffei R , Atene CG , Martinelli S , Potenza L , Luppi M , et al. NOTCH2 contributes to venetoclax resistance in chronic lymphocytic leukemia. Blood. 2019;134(Suppl_1):4280. 10.1182/blood-2019-128499

