# Supplementary material for: Establishment and characterization of a new mantle cell lymphoma cell line with a NOTCH2 mutation, Arbo
Source: EJHaem. 2022 Sep 20;3(4):1326–9. doi: 10.1002/jha2.580 (PMC9713070; doi:10.1002/jha2.580)
Supplement: Supplementary file 1 — Supplement Material [file JHA2-3-1326-s002.pptx]

## Slide 1
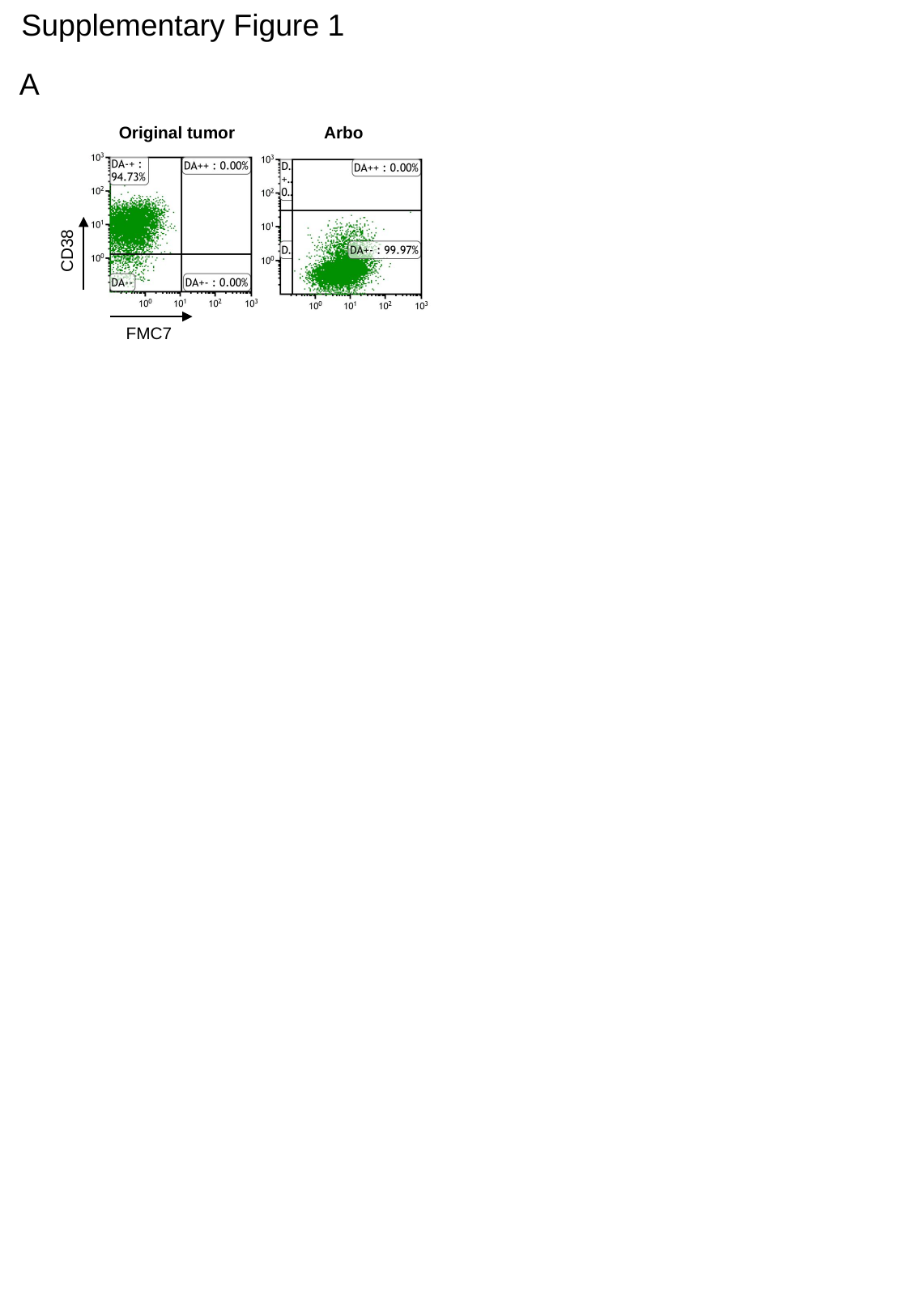

Supplementary Figure 1
A
Original tumor
Arbo
CD38
FMC7

## Slide 2
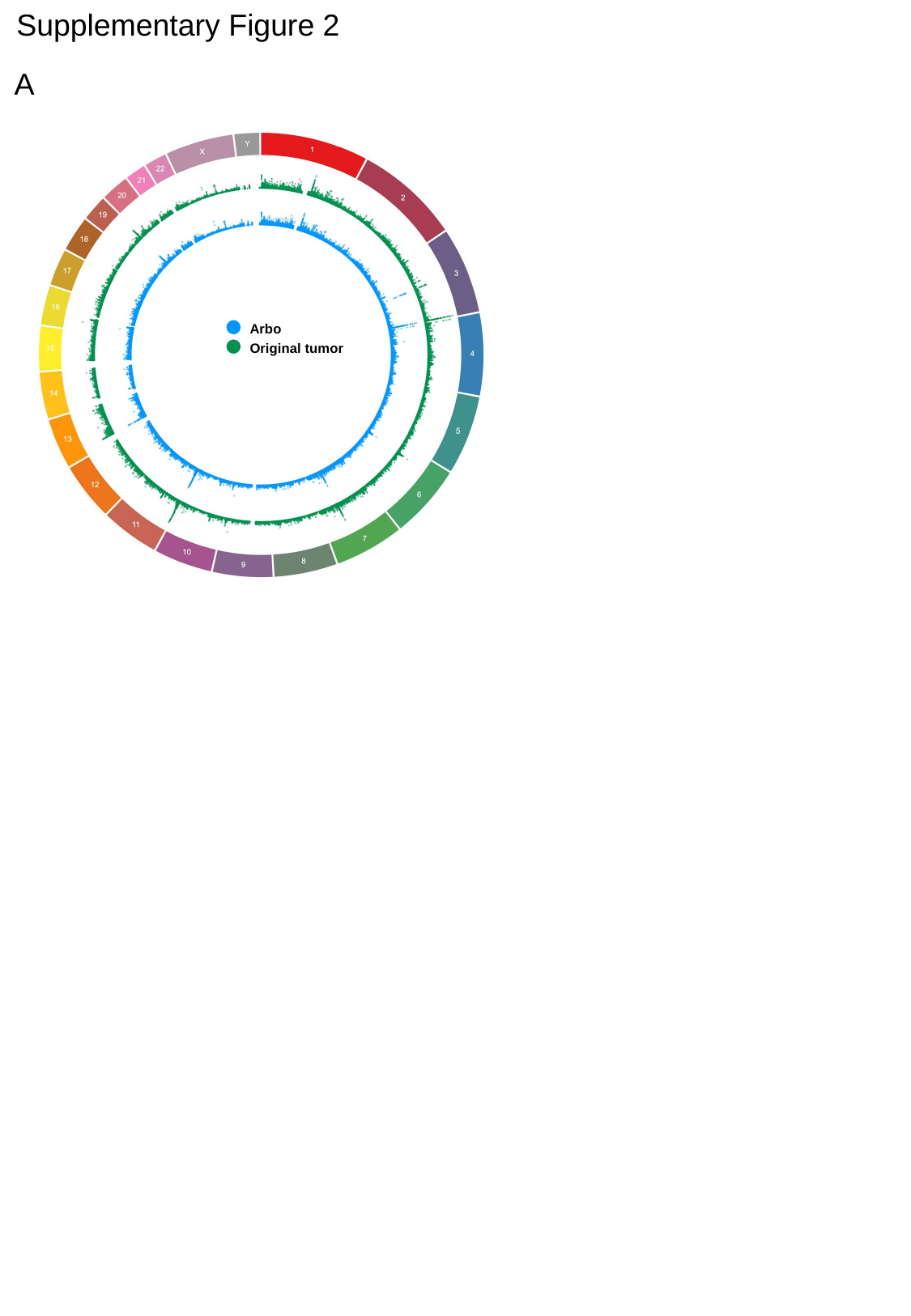

Supplementary Figure 2
A
Arbo
Original tumor

## Slide 3
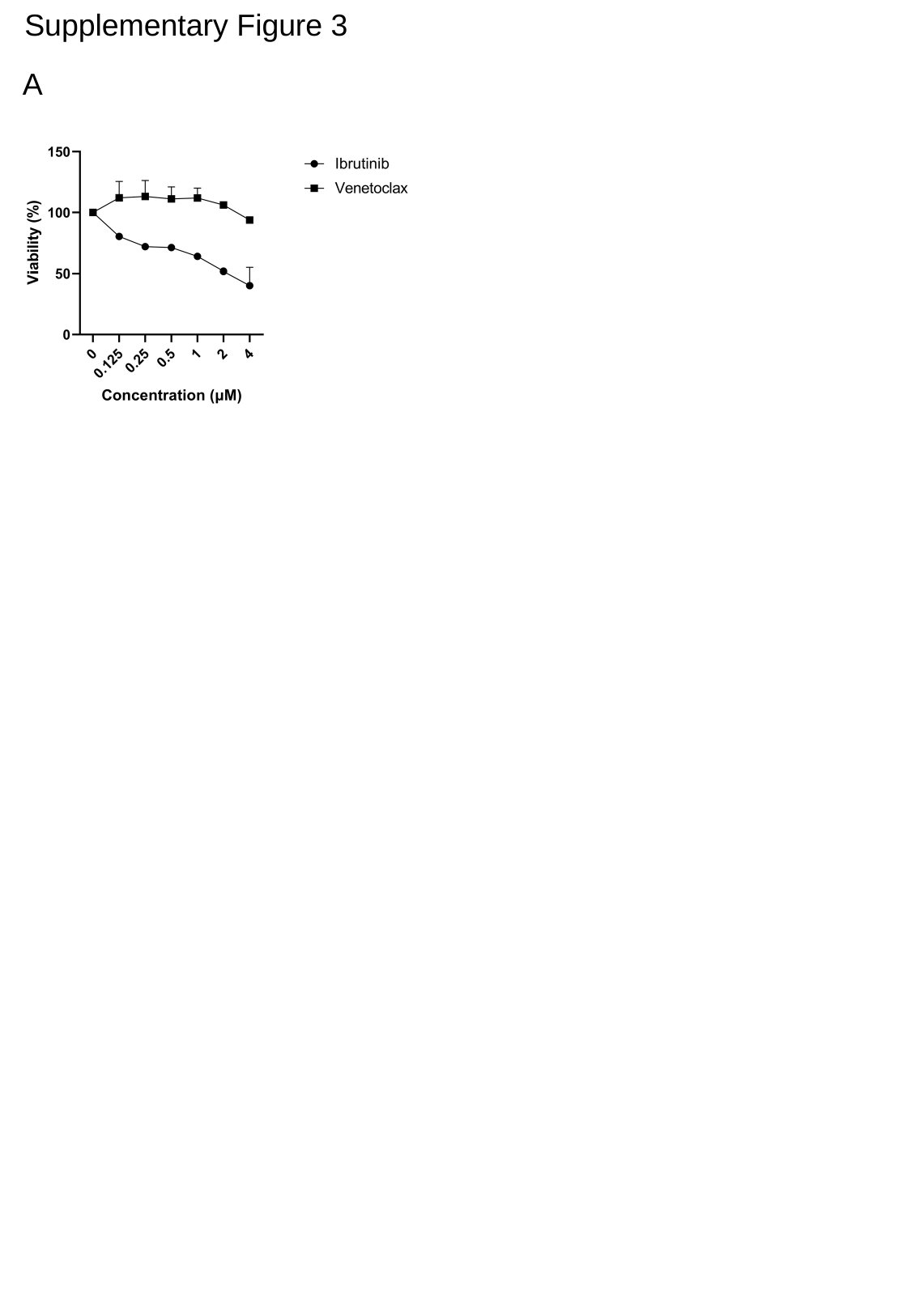

Supplementary Figure 3
A

## Slide 4
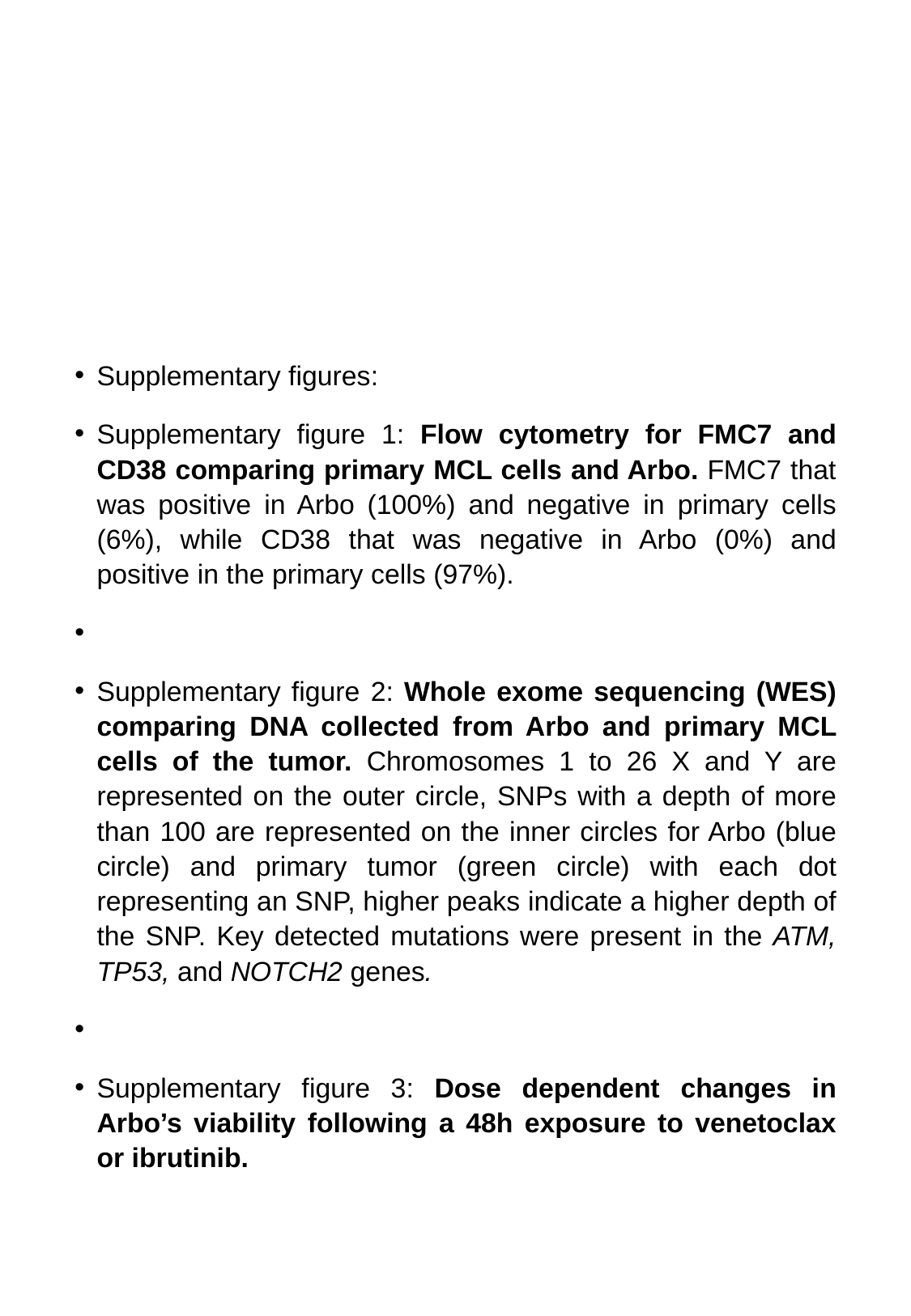

#
Supplementary figures:
Supplementary figure 1: Flow cytometry for FMC7 and CD38 comparing primary MCL cells and Arbo. FMC7 that was positive in Arbo (100%) and negative in primary cells (6%), while CD38 that was negative in Arbo (0%) and positive in the primary cells (97%).
Supplementary figure 2: Whole exome sequencing (WES) comparing DNA collected from Arbo and primary MCL cells of the tumor. Chromosomes 1 to 26 X and Y are represented on the outer circle, SNPs with a depth of more than 100 are represented on the inner circles for Arbo (blue circle) and primary tumor (green circle) with each dot representing an SNP, higher peaks indicate a higher depth of the SNP. Key detected mutations were present in the ATM, TP53, and NOTCH2 genes.
Supplementary figure 3: Dose dependent changes in Arbo’s viability following a 48h exposure to venetoclax or ibrutinib.
